# Feasibility of Nanostructured Lipid Carrier Loaded with Alpha-Mangostin and Clove Oil for Canine Periodontal Therapy

**DOI:** 10.3390/ani14142084

**Published:** 2024-07-17

**Authors:** Gotchagorn Sawatphakdee, Jakarwan Yostawonkul, Saranyou Oontawee, Watchareewan Rodprasert, Chenphop Sawangmake, Chatvadee Kornsuthisopon, Teerapong Yata, Sirinun Pisamai Tabtieang, Nunthawan Nowwarote, Nopadon Pirarat

**Affiliations:** 1 Center of Excellence in Wildlife, Exotic, and Aquatic Animal Pathology, Faculty of Veterinary Science, Chulalongkorn University, Bangkok 10330, Thailand; gotchagorn.sd@gmail.com; 2 National Nanotechnology Center (NANOTEC), National Science and Technology Development Agency (NSTDA), Pathumthani 12120, Thailand; jakarwan@nanotec.or.th; 3 Veterinary Stem Cell and Bioengineering Innovation Center (VSCBIC), Veterinary Pharmacology and Stem Cell Research Laboratory, Faculty of Veterinary Science, Chulalongkorn University, Bangkok 10330, Thailand; saranyou.o@chula.ac.th (S.O.); watchareewan.r@alumni.chula.ac.th (W.R.); chenphop.s@chula.ac.th (C.S.); 4 Veterinary Stem Cell and Bioengineering Research Unit, Faculty of Veterinary Science, Chulalongkorn University, Bangkok 10330, Thailand; 5 Department of Pharmacology, Faculty of Veterinary Science, Chulalongkorn University, Bangkok 10330, Thailand; 6 Center of Excellence in Regenerative Dentistry (CERD), Faculty of Dentistry, Chulalongkorn University, Bangkok 10330, Thailand; 7 Center of Excellence for Dental Stem Cell Biology and Department of Anatomy, Faculty of Dentistry, Chulalongkorn University, 34 Henri-Dunant Rd., Pathumwan, Bangkok 10330, Thailand; chatvadee.k@chula.ac.th; 8 The Biochemistry Unit, Department of Physiology, Faculty of Veterinary Science, Chulalongkorn University, Bangkok, 10330, Thailand; teerapong.yat@mfu.ac.th; 9 Center of Excellence for Companion Animal Cancer, Faculty of Veterinary Science, Chulalongkorn University, Bangkok 10330, Thailand; 10 Department of Veterinary Surgery, Faculty of Veterinary Science, Chulalongkorn University, Bangkok 10330, Thailand; 11 Department of Oral Biology, Faculty of Dentistry, Université Paris Cite, 75006 Paris, France; p_now@hotmail.com

**Keywords:** Alpha-Mangostin, clove oil, canine periodontitis, nanostructured lipid carriers, nanomedicine

## Abstract

**Simple Summary:**

Simple Summary: Periodontitis is a common disease in dogs. While dental scaling and polishing are effective treatments, they come with drawbacks such as anesthetic risks and high costs. To address these challenges, Alpha-Mangostin and clove oil, known for their antibacterial and anti-inflammatory properties, have been used as alternative treatments. Nanotechnology has revolutionized the field of medicine, offering unprecedented advancements in the diagnosis and treatment of various conditions in both humans and animals. As research and development continue to advance, the potential for nanotechnology to address some of the most challenging medical issues, including antimicrobial resistance, is likely to expand, making it an integral component of modern healthcare. This study represents a pioneering effort in developing a pharmaceutical nanostructured lipid carrier spray loaded with Alpha-Mangostin and clove oil as a therapeutic innovation for periodontitis in dogs. The product displayed bacteriostatic efficacy, surpassing certain antibiotic drugs, and high stability. So, its application is valuable in the field of veterinary dentistry.

**Abstract:**

Nanostructured lipid carriers (NLC) represent the second generation of nanoparticles, offering numerous advantages over conventional delivery systems. These include improved stability, enhanced drug-loading capacity, and controlled release profiles, making them highly attractive candidates for a wide range of therapeutic applications. Their suitability for hydrophobic drugs like a traditional medicinal plant of Thailand as clove oil and alpha-mangostin. We investigated into nanostructured lipid carriers loaded with Alpha-Mangostin and clove oil (NLC-AMCO) into the physicochemical and biological characteristics to identify the formulation with the highest efficacy for treatment. The particle size, charge, polydispersity index, and other characterizations were recorded. The realtime ex vivo penetration was explored using canine gingival tissue. Drug sustained release was assessed by HPLC. Moreover, the antibacterial properties were tested by conventional methods. The NLC-AMCO can be stored at up to 40 °C for 60 days without any alterations in particle characteristics. Gingival tissue penetration and sustained drug release were superior compared to unencapsulated counterparts. It exhibited greater effectiveness in inhibiting bacterial growth than the antibiotics tested, particularly against bacteria from the oral cavities of dogs. Therefore, this alternative treatment approach offers cost-effectiveness and ease of administration for pet owners and reduces discomfort for the animals during restraint.

## 1. Introduction

Periodontal disease is the most common disease in small-breed senior dogs, with reported prevalence rates ranging between 80 to 89% in dogs over 3 years of age [1,2,3,4]. This condition primarily arises from bacterial plaque accumulation on tooth surfaces, influenced by multiple factors including age, breed, habits of the dogs, and inadequate oral hygiene practices [5,6]. The canine oral microbiome is a complex microbial community. The shift in bacterial proportions and bacterial accumulation adjacent to the gingival margin potentially lead to gingival inflammation and alveolar bone loss [5,7]. If left unaddressed, uncontrolled periodontal disease can progress to the advanced stage of periodontitis, resulting in tooth loss, pain, and discomfort. Unfortunately, there is a scarcity of medications capable of effectively penetrating the gingival tissues to eradicate bacterial infections.

Recent studies have increasingly emphasized the incorporation of herbal components into contemporary pharmaceutical formulations, aiming to exploit the synergistic effects of plant-based compounds, potentially leading to more effective and less toxic therapeutic options. Notable examples include curcumin, green tea, aloe vera, pomegranate, mangostin, clove, and variegated plants [8,9]. In dentistry, specific interest has been shown in the extracts of Alpha-Mangostin and eugenol extracts, primarily due to their pharmacological properties of being anti-inflammatory and antibacterial against both Gram-positive and Gram-negative bacteria such as *Prevotella intermedia* and *Porphyromonas gingivalis*, as well as *Staphylococcus aureus,* by targeting cell membranes [10,11,12,13,14,15,16]. These bacteria are identified as the keystone pathogens of canine periodontitis. Clinical studies have reported that treatments using these herbal extracts can effectively reduce the pocket depth, decrease the gingival index, and improve clinical epithelial attachment with minimal toxicity [10,17]. Such findings suggest that Alpha-Mangostin and eugenol extracts could be employed as alternatives or adjuncts in the treatment of canine periodontal diseases. Additionally, the anti-biofilm effect of Alpha-Mangostin may be achieved through the inhibition of SarT and IcaB functions [14]. Clove oil exhibits other properties, such as being antifungal, making it effective against infections like Candida, as well as antiviral and analgesic properties used for dental pain [18,19,20,21].

Nanostructured lipid carriers (NLC) represent the second generation of lipid nanoparticles, developed to improve several physicochemical properties such as thermal stability, high drug loading capacity, simplicity of preparation, and cost-effectiveness, along with improving transcellular penetration and drug absorption [22]. These advancements address previous limitations associated with using Alpha-Mangostin and clove oil (AMCO) to treat periodontitis in dogs, such as poor drug bioavailability and rapid clearance by biological fluids, including saliva. The application of NLC loaded with Alpha-Mangostin and clove oil (NLC-AMCO) in the treatment of canine periodontitis has never been explored. This is the first study to investigate the physicochemical properties of NLC-AMCO, as well as its potential to inhibit general bacteria and specifically target keystone pathogens in periodontitis, aiming to provide insights into its applications in veterinary medicine and potentially pave the way for new therapeutic strategies.

## 2. Materials and Methods

### 2.1. NLC-AMCO Preparation

#### 2.1.1. The Oil Phase

**Figure 1:**
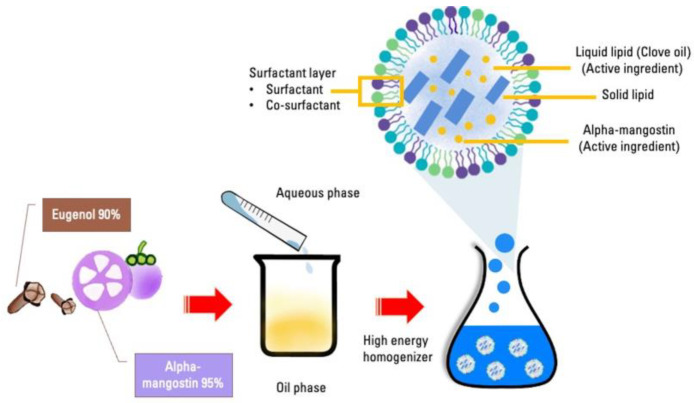
The schematic fabrication and components of nanostructured lipid carrier loaded with Alpha-Mangostin and clove oil (NLC-AMCO).

A lipid phase was formulated by combining 19.75% (*v*/*v*) of clove oil (Krungthepchemi^®^, Bangkok, Thailand) and 0.25% (*w*/*v*) of Alpha-Mangostin (Xi’an Tianguangyuan biological technology, Xi’an, China), extracted from mangosteen peel. Non-ionic surfactants, 2–4% (*v*/*v*) of Span 80 (MySkinRecipes^®^, Bangkok, Thailand) was added. The preparation of the oil-in-water (o/w) emulsion involved the use of 1–3% (*w*/*v*) of MONTANOV^TM^ (Croda^®^, Bangkok, Thailand) as an emulsifier to keep the emulsion well dispersed. To achieve a homogeneous lipid solution, a hotplate magnetic stirrer (RCT basic IKAMAG^®^, Staufen, Germany) was used to stir the mixture for 5 min at a temperature of 25 °C and a speed of 200–400 rpm (Figure 1).

#### 2.1.2. Aqueous Phase

Non-ionic surfactants such as 1–3% (*v*/*v*) of Tween 20^TM^ (Krungthepchemi^®^, Bangkok, Thailand) and 1–3% (*v*/*v*) of glycerol^TM^ (Chemipan^®^, Bangkok, Thailand) were utilized in the preparation process. Subsequently, the 1–3% (*w*/*v*) of Poloxamer 188 solution^TM^ (Croda^®^, Bangkok, Thailand) was added into the solution. These polymers are commonly employed as excipients in pharmaceutical formulations. The solution was mixed and stirred in 68–70% of deionized water (DI-Water) using a hotplate magnetic stirrer (RCT basic IKAMAG^®^, Staufen, Germany) set at 25 °C and a speed ranging between 200–400 rpm for 5 min. The aqueous solution was added to the oil to convert the coarse emulsion into a nanoemulsion using a high-speed homogenizer (T25 digital ULTRA-TURRAX^®^ IKA, Staufen, Germany) at 6000 rpm for 5 min, which facilitated the breakdown of the emulsion into smaller globules.

#### 2.1.3. Formula Fabrication

The study explored varying concentration ratios of Alpha-Mangostin and clove oil including 1:1, 1:2, 1:5, 1:10, 1:50, 1:100, and 1:1000, to identify the optimal ratio that produced a homogenous solution suitable for further processing. Subsequently, the nanoparticles were fabricated by adjusting the surfactant-to-oil ratio (SOR) and hydrophile–lipophile balance (HLB) across ten different formulations. Each formulation was evaluated for physical stability to select the optimal formulation of NLC-AMCO.

### 2.2. Physicochemical Properties of NLC-AMCO

#### 2.2.1. Nanoparticle Characteristics

The characteristics of physical changes, such as color changing and layers separation between oil and water, were recorded on the day of production, day 60, and day 90 post-manufacture, across a variety of temperature settings including 4 °C, 25 °C, 40 °C, and 60 °C.

#### 2.2.2. Stability

Light scattering is a fundamental analytical technique for determining the properties of the nanoparticle. The differences between sample proportions were evaluated by the particle size, zeta potential, and electrophoretic mobility of nanoparticles. The zeta sizer instrument (MALVERN Zetasizer Nano ZSP^®^, Malvern, UK) was used in collecting data day of production, day 60, and day 90 under different temperature conditions including 4° C, 25° C, 40 °C, and 60 °C.

#### 2.2.3. Transmission Electron Microscopy (TEM)

Transmission electron microscopy (JEM-2100 plus, JEOL, Tokyo, Japan) was utilized to observe for the surface morphology of the samples by applying the negative staining technique. The specimen was diluted with a mixture of NLC-AMCO: Type 1 water in the ratio 1:20, followed by staining with 2% phosphotungstic acid (PTA) for 3 min during TEM analysis to improve the contrast and enable observation of the surface morphology.

#### 2.2.4. Kinetic Release of NLC-AMCO

To assess NLC-AMCO, high-performance liquid chromatography (HPLC) was employed to isolate, identify, and quantify each component in a solution. The experiments were conducted to determine the release of clove oil and Alpha-Mangostin from NLC-AMCO.

**Figure 2:**
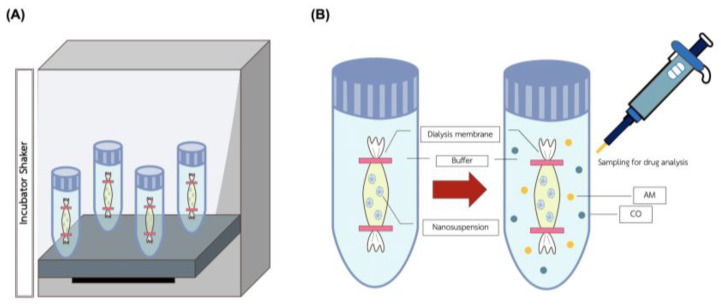
Experimental scheme for the procedure of the NLC-AMCO dialysis membranes. (**A**) Dialysis tube preparation in incubator shaker: NLC-AMCO, Ethanol-AMCO, and DI-AMCO. (**B**) Principle of sample dialysis for in vitro kinetic release testing procedure.

Figure 2 presented information of the sample dilution with ethanol was sonicated by ultrasonic steri-cleaner^®^ (Sturdy Co., Ltd., Waltrop, Germany) for 30 min, and filtered both parts of the sample through a 0.20 µm Nylon membrane filter (Thermo Scientific^®^, Cambridge, MA, USA) to measure the total concentration. The NLC-AMCO was separated between entrapped drug and free drug by centrifugation (MPW-352R, Warsaw, Poland) at a speed of 18,000 RPM for 60 min. Then, samples were collected from the indirect solution and directly delivered to the HPLC for evaluating the encapsulation efficiency. The release of NLC-AMCO, Ethanol-AMCO, and insoluble DI-AMCO were compared in the same conditions.

The HPLC method was performed on a Shimadzu CBM-20A HPLC system controller (Shimadzu Corporation, Kyoto, Japan), equipped with a model LC-20AD pump, Degassing unit DGU-20A3R, UV detector SPD-20AV, Autosampler model SIL-20A with a 100 µL loop, and column oven CTO-10AS VP. A Shim-pack VP-ODS HPLC column^®^ (250 L × 4.6) with a Shim-pack GVP-ODS 10 L × 4.6 guard column were used. The elution was carried out with isocratic solvent systems with a flow rate of 1.5 mL/min at a temperature of 40 °C. The mobile phase consisted of 2% acetic acid (RCI Labscan Ltd., Bangkok, Thailand) in DI water (solvent A) and acetonitrile HPLC grade (RCI Labscan Ltd., Bangkok, Thailand) (solvent B). The mobile phase was prepared daily, filtered through 0.45 µm of glass microfiber filters (Whatman™, Kent, UK), by a vacuum pump (Vacuubrand^®^ ME 1C., Wertheim, Germany), and sonicated before use. The total running time was 15 min. Clove oil was prepared at a constant ratio of 50% acetonitrile and 50% acetic acid. In contrast, Alpha-Mangostin has a constant ratio of 80% acetonitrile and 20% acetic acid, modified from the protocol of Chaivisuthangkura et al. [23]. There was 15 min of postrun for reconditioning. The sample injection volume used 10 µL while the wavelength of the UV-vis detector was set at 320 nm for Alpha-Mangostin and 254 nm for clove oil. The relative amounts of each component were calculated with peak areas using LabSolutions software version 5.109.

#### 2.2.5. Drug Loading and Release from NLC-AMCO

The percentage of drug loading and entrapment efficiency were calculated using the following formula [24,25]:

Encapsulation efficiency (%) = (Amount of drug released from NLC-AMCO/Amount of drug initially taken to prepare the NLC-AMCO) × 100

Drug loading (%) = (Amount of drug found in the NLC-AMCO/Amount of NLC-AMCO) × 100

#### 2.2.6. Realtime Ex Vivo Canine Gingiva Penetration

All samples were collected from Small Animal Hospital, Faculty of Veterinary Science, Chulalongkorn University. The animal ethics guidelines were approved by the Animal Ethics Committee, Chulalongkorn University (CUACUC; Approval No. 2331042). The canine gingival tissues were harvested from a healthy and periodontitis dog in the maxillary and mandibular regions, each measuring approximately 3 × 3 × 0.5 mm by dissection using a sterile scalpel blade No. 11. A total of 14 full-thickness gingival tissues were prepared from 6 fresh carcasses within 4 h post-mortem. The samples were stored at 4 °C overnight in 1 mL phosphate-buffered saline (PBS). Tissue penetration assays were conducted within a maximum of 24 h post-mortem, adhering to the guidelines previously published [26].

The gingival tissues were divided into 3 groups: (1) Nile red fluorescent with normal saline solution (NR-NSS), (2) Nile red fluorescent with nanostructured lipid carriers loaded with Alpha-Mangostin combination with clove oil (NR-NLC-AMCO), and (3) Nile red fluorescent with Alpha-Mangostin combination with clove oil (NR-AMCO). For each group, 10 µL of the respective emulsion was applied onto freshly prepared gingival tissue for 5 min at room temperature in dark environment. Subsequently, the sample were washed 3 times with PBS to remove excess emulsion. The tissue was placed on the surface of a spacer measuring 15 × 15 × 0.5 mm that was attached to a sterile slide. A 5 µL drop of mounting solution was applied to the tissue. A coverslip was then gently pressed onto the sample to ensure uniform contact and distribution [27].

**Figure 3:**
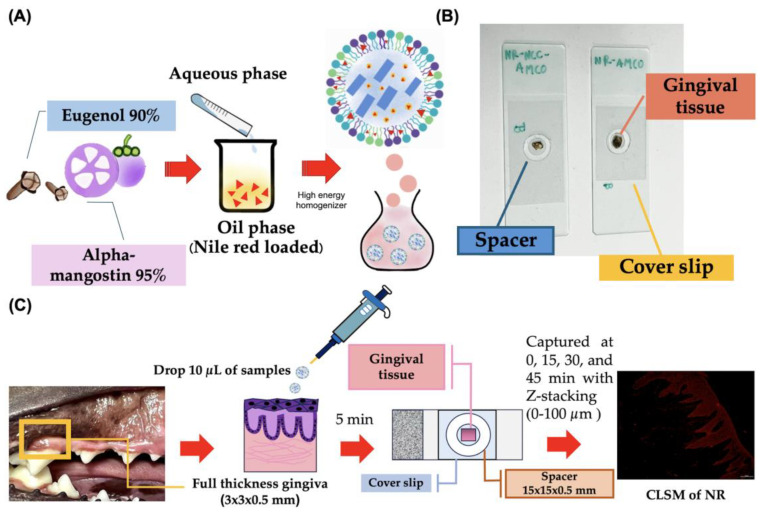
Illustration of the realtime ex vivo canine gingiva penetration assay. (**A**) A step-by-step for mixing Nile red fluorescence stain with NLC-AMCO. (**B**) Preparation of the sample for confocal fluorescence microscope. (**C**) Schematic diagram of the canine gingival penetration test.

Images were sequentially stacked in real time every 4 µm from bottom to top view using Nikon AX/AX R Confocal Microscope, with a measuring range of 0 to 100 µm at intervals of 0, 15, 30, and 45 min under various conditions (Figure 3). The transgingival fluorescence signals were recorded by NIS-Elements AR (Advanced Research) software version 5.42.00. Each condition was performed in triplicate [28].

The relative penetration rate of Nile red intensity (NRI; Ex: 561 nm, Em: 571–637 nm, 200× magnification, FOV: 880.64 µm) was calculated using initial time point normalization, defined as NRIP = NRIt/NRI0 × 100, where NRIt is the intensity at a given time point and NRI0 is the baseline intensity.

#### 2.2.7. Antibacterial Properties

The disc diffusion method was performed following Kirby–Bauer disk diffusion susceptibility test protocol [29] to determine the antibacterial activity of the NLC-AMCO. The most common strains in drug discovery for quality control included *Staphylococcus aureus* (ATCC 29213), *Staphylococcus aureus* (ATCC 25923), *Escherichia coli* (ATCC 25922), *Escherichia coli* (ATCC 35218), *Pseudomonas aeruginosa* (ATCC 27853), and *Streptococcus pneumoniae* (ATCC 49619). *Bacteroides pyogenes* and *Klebsiella Pneumoniae* were isolated from subgingival areas of the oral cavity in dog for comparative analyses. The antimicrobial efficacy of NLC-AMCO, AMCO, Alpha-Mangostin (AM), clove oil (CO), and the first-line antibiotics commonly used to treat periodontitis in dogs was assessed. Standardized concentrations of antimicrobial agents were employed, including amoxicillin 10 μg (CT0161B—OXOID Ltd., Hampshire, UK), doxycycline 30 μg (CT0018B—OXOID Ltd., Hampshire, UK), clindamycin 2 μg (CT0064B—OXOID Ltd., Hampshire, UK), and metronidazole 5 μg (CT0067B—OXOID Ltd., Hampshire, UK). These were placed on top of an agar surface, and the inhibition zone around each disk were measured after overnight incubation by electronic digital vernier caliper (SINAT™, Zhenjiang Co., Ltd., Zhenjiang, China).

### 2.3. Statistical Analysis

The results were presented in means ± standard error (SE). The statistical analyses were performed utilizing R-Studio software version 2023.06.1 (R Core Team, 2023). The *t*-test was used for comparisons, while one-way ANOVA was employed for analyses involving multiple groups. For nonparametric data, the Wilcoxon matched-pairs signed-rank test was considered for two-group comparison. The Kruskal–Wallis test was used for multiple group comparisons followed by Dunn’s test as a post-hoc pairwise comparison. A value of *p* < 0.05 was considered statistically significant.

## 3. Results

### 3.1. Physical Characteristics of NLC-AMCO

The NLC-AMCO proportion was selected at 1:100, representing the minimum concentration that could be effective to inhibit bacteria while also being suitable for forming a homogenous solution (Figure S1).

The experiment explored the physical characteristics of NLC-AMCO under varying temperatures. The formulation remained unchanged and appeared as a milky-whitish, homogenized dispersion for up to 90 days at temperatures of 4 °C, 25 °C, and 40 °C.

However, at 60 °C, significant changes were observed after 30 days, including phase separation between the oil and aqueous layers, along with alterations in color and odor (Figure S2).

### 3.2. Stability of NLC-AMCO

**Figure 4:**
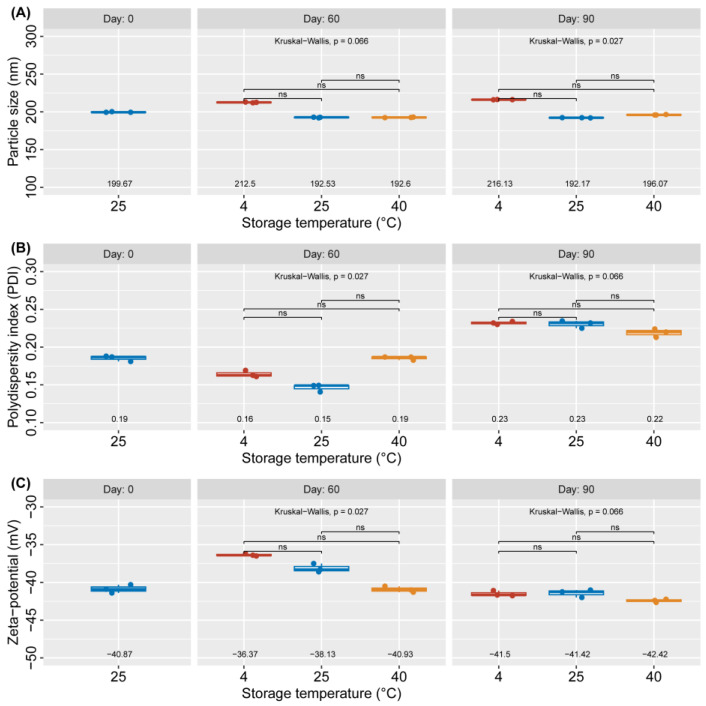
The particle stability of NLC-AMCO after incubation at 4, 25 and 40 °C on days of production, day 60, and day 90, based on (**A**) particle size, (**B**) polydispersity index, and (**C**) zeta-potential.

The stability of NLC-AMCO was assessed on day of production, day 60, and day 90 using a zeta sizer instrument, with results presented in Figure 4. There were no statistically significant differences in the average particle size of NLC-AMCO at 60 and 90 days. Particles sizes ranged from 212.50 to 192.53 nm at 60 days and from 216.13 to 196.07 at 90 days under various storage temperature were used in this study. The dispersity of particles in an emulsion remained below 0.23. The zeta potential of the NLC-AMCO was consistently less negative charge than −42.42 mV.

### 3.3. Transmission Electron Microscopy (TEM)

**Figure 5:**
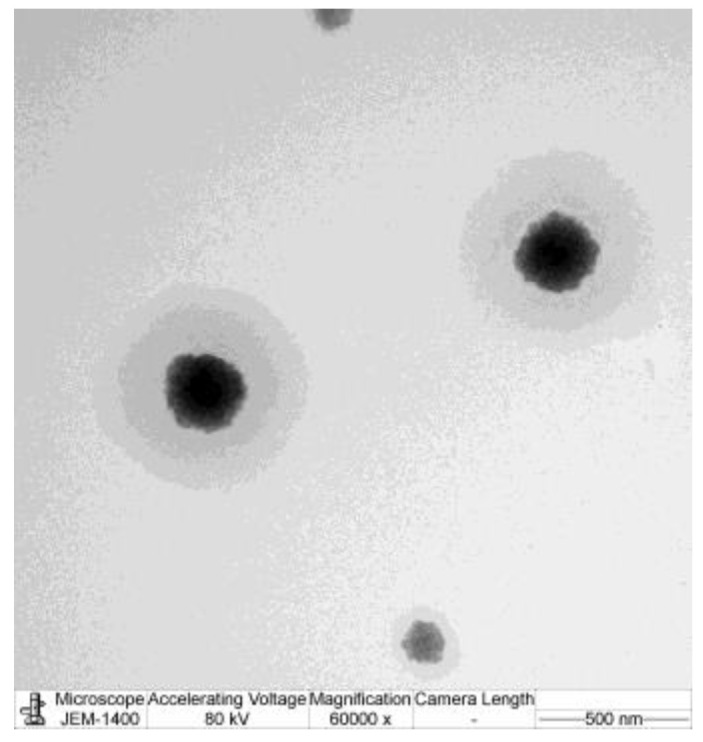
Transmission electron micrograph of NLC-AMCO (scale bar: 500 nm).

The TEM image in Figure 5 revealed that the particles of NLC-AMCO were spherical and predominantly around 200 nm in diameter, which corelated with the particle size results obtained from the dynamic light scattering. The NLC-AMCO particles were monodispersed and exhibited a uniform size distribution.

### 3.4. Kinetic Release of NLC-AMCO

**Figure 6:**
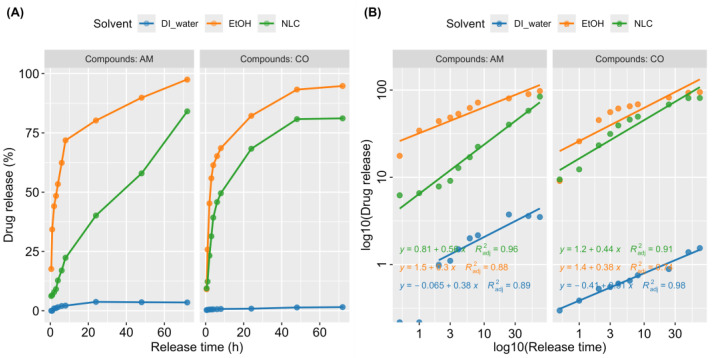
The schematic representation drug release of NLC-AMCO. (**A**) Scatter plots with drug release profile of Alpha-Mangostin (AM), and clove oil (CO) with various carriers; nanostructured lipid carrier (NLC), ethanol (EtOH), and deionized water (DI water). (**B**) Scatter plots with linear regression for visualization of release kinetic of Alpha-Mangostin (AM), and clove oil (CO) with various carriers; nanostructured lipid carrier (NLC), ethanol (EtOH), and deionized water (DI water).

The cumulative drug release profiles of AMCO were observed via the percentage of AMCO released relative to the amount NLC-AMCO and compared with control in DI-water and EtOH. As illustrated in Figure 6, in vitro drug release kinetics studies indicated that 25% of AMCO was released from NLC within 8 h, followed by a sustained release over a period of 72 h. In contrast, AMCO exhibited low water solubility, resulting in minimal excretion in DI-water. In addition, our study discovered that AMCO in EtOH showed an initial kinetic burst release within the first 8 h. The Korsmeyer–Peppas model in Table 1 was used to describe and analyze the release of a drug from NLC-AMCO, the n value was applied to characterize the AMCO release mechanism. The NLC-AM is non-quasi-Fickian diffusion; however, NLC-CO and other formulations followed quasi-Fickian diffusion. Of all solvent conditions tested, the release of both AM and CO from NLC formulations fitted well with correlation coefficients (R^2^ values) of 0.96 and 0.91, respectively. The release constant (k value) indicated that the release rate of AMCO in EtOH was faster than that from NLC, and the release from NLC was more rapid compared to that in DI water.

### 3.5. Drug Loading and Release from NLC-AMCO

The average encapsulation efficiency of AM was 98.66% and 98.95% in CO. Drug loading capacity was 0.2% in AM and 20% in CO.

### 3.6. Realtime Ex Vivo Canine Gingiva Penetration

**Figure 7:**
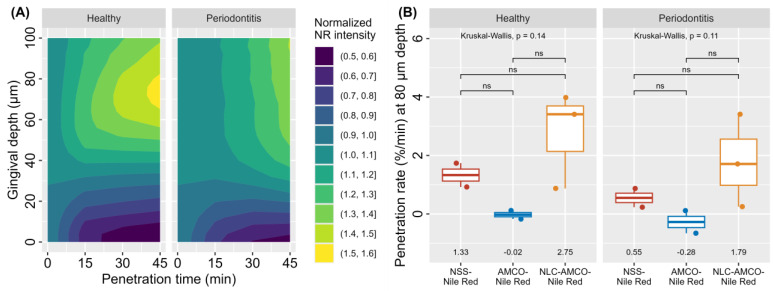
Schematic representation of the canine gingival penetration. (**A**) Contour plot of gingival depth and time: lighter colors indicated higher NR intensity, i.e., greater penetration associated gingival depth. (**B**) Box plots of relative gingival penetration rate in healthy and periodontitis dogs under different conditions of treatment: NSS, AMCO, and NLC-AMCO.

According to the results presented in Figure 7, the two main factors influencing gingival penetration rate were substance type and gingival characteristics. However, no statistically significant differences were observed in gingival characterization for NLC-AMCO. Interestingly, NLC-AMCO demonstrated enhanced penetration compared to AMCO and NSS in both healthy and periodontitis-affected dogs.

**Figure 8:**
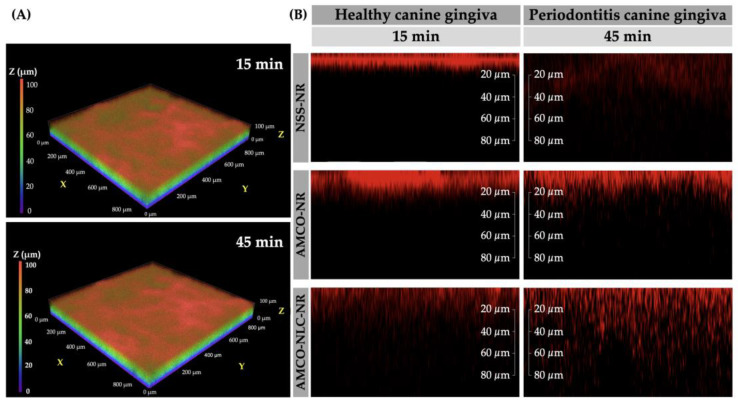
Three-dimensional CLSM micrographs of penetration and accumulation of NSS-NR, AMCO-NR, and AMCO-NLC-NR in healthy and periodontitis canine gingiva. (**A**) Illustrations of 3D CLSM micrographs after penetration and accumulation of AMCO-NLC-NR into periodontitis canine gingiva at various time intervals. (**B**) Comparative analysis of NR accumulation within each layer (20–80 µm) under different conditions following a 45 min exposure.

Three-dimensional confocal light scanning microscopy (3D-CLSM) revealed that topical exposure of AMCO-NLC-NR achieved better gingival epithelium penetration compared to NSS-NR and AMCO-NR (Figure 8). Post-application, NR staining showed increased penetration into the epithelium layer, with concurrent decreased penetration into the lamina propria.

### 3.7. Antibacterial Activity of NLC-AMCO

**Figure 9:**
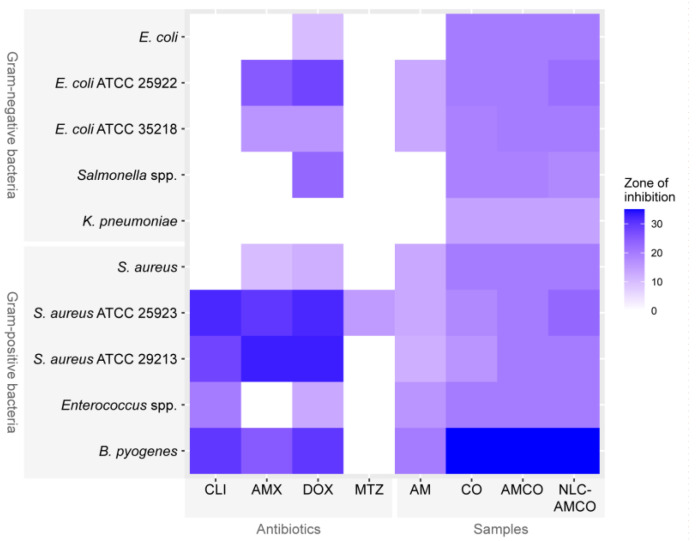
Heat map analysis illustrating the zone of inhibition of the first-line antibiotics used to treat periodontitis in dogs: CLI: clindamycin (2 µg); AMX: Amoxicillin (10 µg); DOX: Doxycycline (30 µg); MTZ: Metronidazole (5 µg) and samples (20 µL); AM: Alpha-Mangostin (2000 ppm); CO: clove oil (20%); AMCO: Alpha-Mangostin combination with clove oil; NLC-AMCO: nanostructured lipid carrier loaded AMCO. All sorts of color density in the figure demonstrate inhibiting zone of bacteria.

The disc diffusion test shown in Figure 9 demonstrates the inhibiting zone of samples including AM, CO, AMCO, and NLC-AMCO compared to standard antibiotics. The Gram-negative bacteria tested, including *E. coli*, *Salmonella* spp. and *Klebsiella Pneumoniae*, exhibited resistance to clindamycin, amoxicillin, and metronidazole. On the contrary, CO, AMCO, and NLC-AMCO showed inhibitory zones, suggesting their ability to inhibit bacterial growth. Specifically, while both *Escherichia coli* (ATCC 25922) and *Escherichia coli* (ATCC 35218) indicated resistance to clindamycin and metronidazole (Figure S3). But, all the samples of AM, CO, AMCO, and NLC-AMCO were able to inhibit the growth of these bacteria. The NLC-AMCO exhibited greater effectiveness in inhibiting bacterial growth than the antibiotics tested, particularly against *Bacteroides pyogenes* and *Klebsiella Pneumoniae*, which were isolated from the oral cavity of dogs.

## 4. Discussion

NLC represents the second generation of lipid-based nanoparticles, offering advancements over their predecessors, including liposomes, nanoemulsions, and solid lipid nanoparticles (SLNs). These advancements are distinguished by enhanced stability, increased drug loading capacity, and improved safety for tissue applications [30,31]. Despite the growing interest in nano innovations, their potential in veterinary drug delivery remains largely underexplored.

The current study is part of an initiative to leverage medicinal herbs for therapeutic applications in the veterinary field, aiming to enhance the commercial value of agricultural products in Thailand. This approach is motivated by the rational use of drugs and the need to address the limitations posed by antibiotic resistance. Consequently, Alpha-Mangostin and clove oil were selected for their intrinsic properties. This research marks the first published investigation into the combined efficacy of Alpha-Mangostin and clove oil, thus contributing novel insights into their potential synergistic effects.

NLC-AMCO was achieved to synthesis and investigation its physicochemical and biological properties, notably morphological characterization, stability, penetration, drug release, and antibacterial activity. The NLC-AMCO can be stored for up to 60 days at temperatures not exceeding 40 °C without statistically significant changes in its properties. This formulation exhibited a highly negative zeta potential (up to −30 mV), which is indicative of its high stability in solution. In addition, the average particle diameters were less than 200 nm and the PDI remained between 0.1 and 0.2, signifying a monodisperse distribution of NLC-AMCO particles [32,33].

Interestingly, the results showed that NLC-AMCO exhibited substantial bacteriostatic activity against multidrug-resistant (MDR) and extensively drug-resistant (XDR) bacteria include *E. coli*, *K. pneumoniae*, *S. aureus*, which have been previously reported in humans [34,35]. Previous research has shown that Alpha-Mangostin is effective against bacteria by increasing cell membrane permeability. However, it remains unclear which specific genes or proteins are involved in this process [13,14]. The major antibacterial mechanism of clove oil is the disruption of the cell membrane structure, leading to the leakage of intracellular contents and resulting in cell death [16,36]. The inhibitory zones of clove oil, aligning with previous finding [37], were 24 mm for *S. aureus* and 21.9 mm for *E. coli*. Similarly, Alpha-Mangostin exhibited zones of inhibition measuring 13 mm against *S. aureus* and 10 mm against *E. coli* [38]. The combination of AM and CO did not interfere with their antibacterial effectiveness. In addition, the nanoparticle formulation preserved the antibacterial properties without any detriment. In contrast, the NLC system improved the stability of AMCO and facilitated a sustained release of the drug up to 72 h. This is consistent with previous studies on AM, which demonstrated an initial release of 25% during the first 10 h and sustained release for at least 50 h [32]. However, the nanoparticle exhibited limited water solubility for AM and a significant burst release, achieving 90% release within 25 h.

This study represents the first publication to utilize the ex vivo canine gingival model for real-time gingival penetration assay of NR by CLSM. This method offers advantages over traditional techniques, including cryostat sectioning and staining, by allowing for real-time observation and easier examination of samples, in addition to reducing fluorophore fading in tissue samples. Our results showed that NLC-AMCO effectively penetrated through the stratum corneum, reaching depths of at least 100 µm within 45 min in both healthy and periodontitis-affected gingival tissues. This level of penetration aligns with previous findings that reported penetration depths ranging from 30–90 µm in the stratum corneum of human skin using N-methyl-2-pyrrolidone (NMP) niosomes [39]. The predominant mechanisms for oral mucosal drug penetration were the transcellular and paracellular pathways [40]. NLC-AMCO predominantly utilized the paracellular pathway for penetration, enabling diffusion between cells. This mechanism allowed NLC-AMCO to achieve superior penetration compared to AMCO, facilitated by the small size of the nanoparticles [30]. The sample preparation in the current study was consistent with prior ex vivo research, which involved delivering vitamin E acetate and vitamin F from toothpaste to porcine gingival tissue using liquid scintillation counting to detect radioactivity of both vitamins in viable layers [26]. However, instead of radioactive compounds, the current study used fluorescence tagging with NR, following methodologies on the penetration of NLC in porcine cornea [41]. The advantages of using NR were enhanced by its widespread application for fluorescent lipid staining in various cell types, including smooth muscle cells and macrophages, and its high sensitivity for lipid staining in tissue sections [42,43,44]. Moreover, the wavelength of NR fluorescence was not disturbed by autofluorescence, a crucial consideration in experimental design and result interpretation [45].

This study encountered several limitations, primarily stemming from the small sample size used in the penetration test, which impacted the ability to establish statistically significant differences between groups. To enhance accuracy, it is advisable to select gingival tissues devoid of melanin pigmentation and subgingival plaque to minimize interference from light absorption during fluorescence intensity measurements. The decision to limit the experimental depth to 100 µm was reasoned by the practical consideration that exceeding this depth might hinder the detection of NLC-AMCO, potentially due to substance diffusion into other tissue layers. Furthermore, the frequency of high-power laser shots has been observed to influence the color fading of NR fluorescence, resulting in diminished signals in deeper layers.

Alpha-Mangostin and clove oil, the active components of NLC-AMCO, are characterized by low water solubility. This limitation can be effectively overcome through nanoencapsulation to enhance their therapeutic properties. This study represents the first to document the efficacy of NLC-AMCO, suggesting its potential application in the field of veterinary dentistry and beyond, due to the broad-spectrum benefits conferred by its formulation. Moreover, NLC-AMCO could be formulated into various delivery systems suitable for addressing bacterial infections in different clinical settings, such as wound management. The issue of antimicrobial resistance (AMR) in zoonotic pathogens, which affects both animals and humans, particularly in livestock, is considered as a major public health challenge. Therefore, the application of NLC-AMCO across multiple species, including aquatic animals, ruminants, wildlife, and exotic species, could offer a novel modality for managing infections caused by multidrug-resistant organisms and also provide economic benefits.

Alpha-Mangostin and eugenol possess anti-inflammatory and anti-biofilm formation capabilities [12,16,46]. Further studies with NLC-AMCO will be required to confirm these findings. Given the significant antimicrobial activity demonstrated by NLC-AMCO against *Bacteroides pyogenes* and *Klebsiella Pneumoniae*, pathogens associated with periodontitis in dogs [47,48,49], it would be beneficial to extend these studies to other bacteria commonly implicated in periodontal diseases.

## 5. Conclusions

The pharmaceutical archetype of NLC-AMCO spray represents an alternative treatment strategy for attenuating a broad spectrum of bacteria, including both Gram-positive and Gram-negative bacteria. The small particle size and enhanced water solubility of NLC-AMCO contribute to its increased efficiency in penetration through gingival tissues. Additionally, NLC-AMCO demonstrates favorable biostability and sustained drug release properties, enabling stable storage at up to 40 °C for 60 days.

## Figures and Tables

**Table 1:** The Korsmeyer–Peppas model for determination of the diffusion characteristics of AM and CO from with various carriers; NLC, EtOH, and DI water.

Active Compounds	Solvent	b (Y-Intercept)	K (10^b^)	n (Slope)	R^2^
AM	NLC	0.81	6.46	0.56	0.96
AM	EtOH	1.5	31.62	0.3	0.88
AM	DI water	−0.065	0.86	0.38	0.89
CO	NLC	1.2	15.85	0.44	0.91
CO	EtOH	1.4	25.12	0.38	0.74
CO	DI water	−0.41	0.39	0.31	0.98

b is the y-intercept, K is the transport constant, n is the transport exponent, R^2^ is the coefficient of determinations.

## Data Availability

The original contributions presented in the study are included in the article/supplementary material, further inquiries can be directed to the corresponding author/s.

## Supplementary Materials

The following supporting information can be downloaded at: https://www.mdpi.com/article/10.3390/ani14142084/s1, Figure S1: The proportional ratio of Alpha-Mangostin and clove oil in each dilution as 1:1, 1:2, 1:5, 1: 10, 1:50, 1:100, 1:1000, and 1:2000. The dilution that homogenous since 1:50 (A) and the efficiency of the antibacterial properties test in *Staphylococcus aureus* (ATCC 29213) and *Escherichia coli* (ATCC 35218) was not different (B). Figure S2: Physiological characterization of NLC-AMCO in 60 days (A) and 90 days (B) under various temperature as 4 °C, 25 °C, 40 °C, and 60 °C. Figure S3: Kirby-Beauer disc diffusion susceptibility test protocol. Both *Escherichia coli* (ATCC 25922) (A) and *Escherichia coli* (ATCC 35218) (B) resisted to clindamycin (CLI) and metronidazole (MTZ).
