# Supplementary material for: Feasibility of Nanostructured Lipid Carrier Loaded with Alpha-Mangostin and Clove Oil for Canine Periodontal Therapy
Source: Animals (Basel). 2024 Jul 17;14(14):2084. doi: 10.3390/ani14142084 (PMC11273492; doi:10.3390/ani14142084)
Supplement: Supplementary file 1 [file animals-14-02084-s001.zip › animals-3093697-supplementary.pdf]

Supplementery file

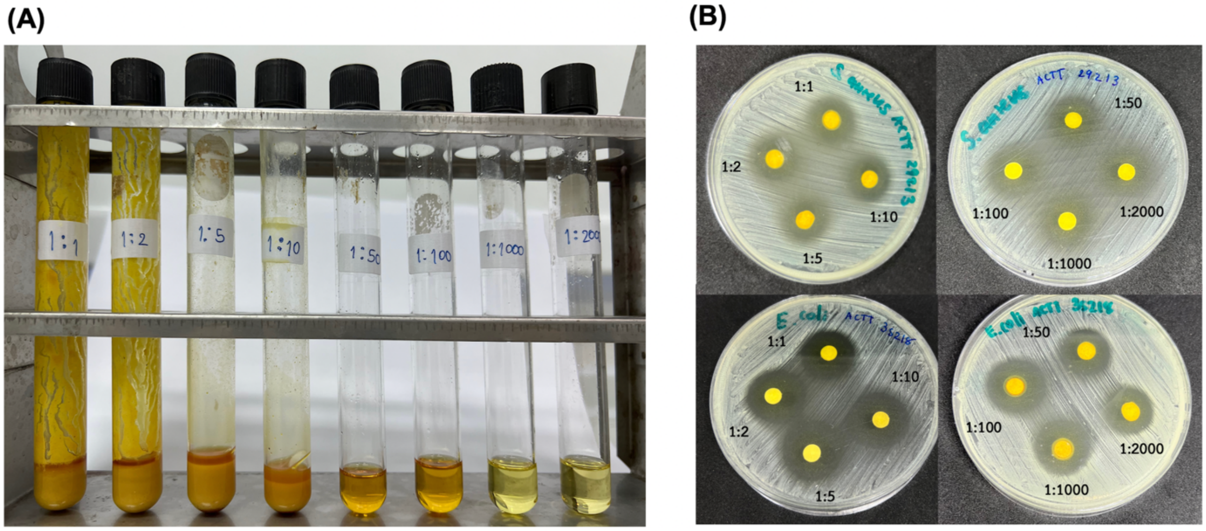

Figure S1: The proportional ratio of Alpha−Mangostin and clove oil in each dilution as 1:1, 1:2, 1:5, 1: 10, 1:50, 1:100, 1:1000, and 1: 2000. The dilution that homogenous since 1:50 (A) and the efficiency of the antibacterial properties test in *Staphylococcus aureus* (ATCC 29213) and *Escherichia coli* (ATCC 35218) was not different (B).

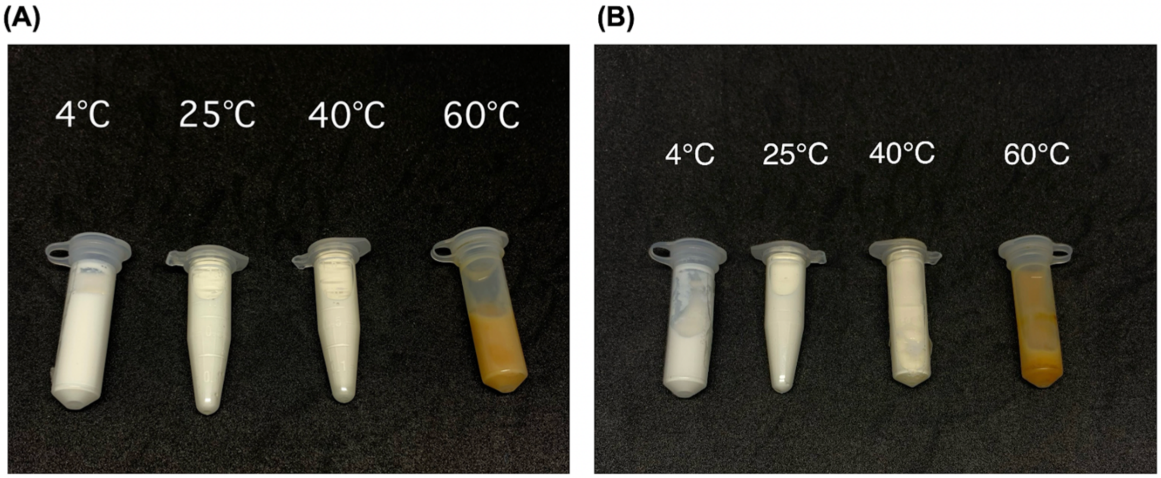

Figure S2: Physiological characterization of NLC−AMCO in 60 days (A) and 90 days (B) under various temperature as 4 °C, 25 °C, 40 °C, and 60 °C

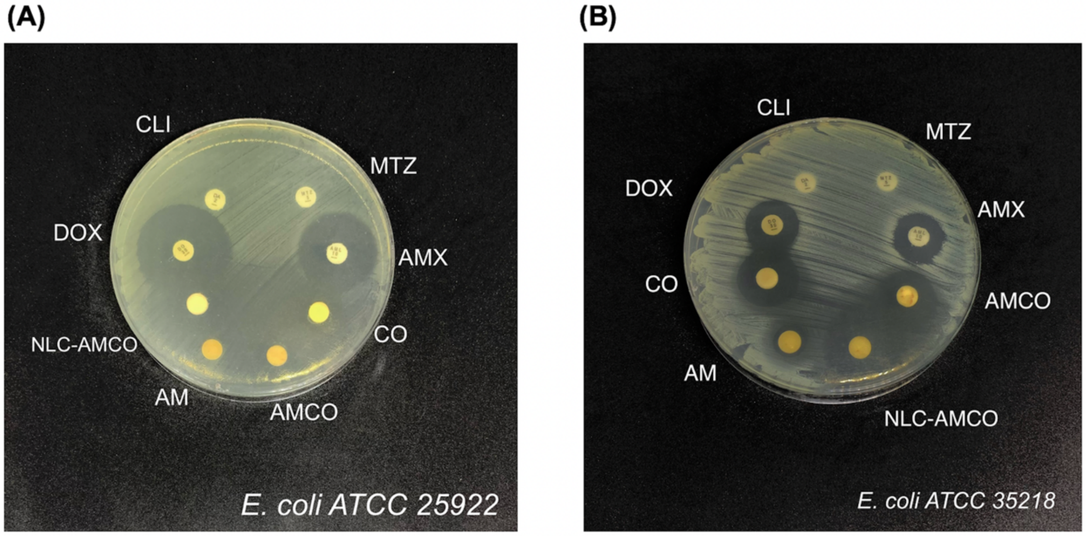

Figure S3: Kirby–Beauer disc diffusion susceptibility test protocol. Both *Escherichia coli* (ATCC 25922) (A) and *Escherichia coli* (ATCC 35218) (B) resisted to clindamycin (CLI) and metronidazole (MTZ).
